# Optical coherence tomography-guided decision-making for Descemet stripping automated endothelial keratoplasty in Ahmed glaucoma valve-related corneal decompensation

**DOI:** 10.22336/rjo.2026.36

**Published:** 2026

**Authors:** Vijay Kumar Sharma, Prabhjot Singh, Gaurav Kapoor, Vikas Ambiya, Sagrika Patyal

**Affiliations:** 1 Department of Ophthalmology, Armed Forces Medical College, Pune, India; 2 Centre for Sight, New Delhi, India

**Keywords:** Ahmed glaucoma valve, corneal decompensation, anterior segment OCT, tube tip to endothelium distance, Descemet stripping automated endothelial keratoplasty, AGV = Ahmed glaucoma valve, AS-OCT/ASOCT = Anterior segment optical coherence tomography, Aug Trab = Augmented Trabeculectomy, BCVA = Best-corrected visual acuity, CFCF = Counting fingers close to face, HD-OCT = High-definition optical coherence tomography, HMCF = Hand movements close to face, IOP = Intraocular pressure, mmHg = Millimeters of mercury, OCT = Optical coherence tomography, SER = Serial number, TTE = Tube tip to endothelium, T-C angle = Tube corneal angle, T-UCVA = Uncorrected visual acuity, μm = Micrometer

## Abstract

**Objective:**

Ahmed glaucoma valve (AGV) implantation can lead to corneal decompensation in a significant proportion of patients. Anterior segment optical coherence tomography (ASOCT) evaluation may serve as a crucial tool for assessing AGV retention versus explantation and for planning Descemet stripping automated endothelial keratoplasty (DSAEK) in such cases. This study aims to evaluate the utility of ASOCT in guiding management decisions for AGV-related corneal decompensation.

**Materials and methods:**

In this prospective interventional study, 11 eyes with AGV-related corneal decompensation were evaluated using anterior segment 5-line raster-mode OCT measurements. Parameters assessed included stromal scarring, corneal thickness, and tube tip to endothelium (TTE) distance. ASOCT findings guided management decisions: four patients with nonfunctional AGVs underwent staged procedures (AGV removal with augmented trabeculectomy, followed by DSAEK), while seven patients underwent DSAEK alone (six with functional AGVs with TTE distance > 150 μm; one patient with a non-functional AGV refused consent for AGV explantation).

**Results:**

All patients demonstrated significant improvement in best-corrected visual acuity with well-controlled intraocular pressure. Graft attachment was successful in all cases except one patient who experienced detachment after one month. Based on our findings, we proposed a protocol for managing AGV-related corneal decompensation.

**Discussion:**

Tube shunt implantation effectively reduces intraocular pressure in eyes with uncontrolled glaucoma but can lead to significant endothelial cell loss and corneal decompensation. In this series, two major challenges were addressed: the decision regarding AGV tube removal and its risks, and the determination of a safe tube-tip-to-endothelium (TTE) distance to prevent graft damage after DSAEK. We considered a preoperative TTE greater than 150 μm to be safe for single-stage DSAEK without AGV modification, accounting for lenticule thickness and the anticipated reduction in corneal edema. ASOCT-guided measurement of TTE distance proved invaluable for predicting complications and guiding surgical decision-making, with outcomes that support our proposed management protocol.

**Conclusion:**

While performing DSAEK in patients with AGV-related corneal decompensation presents significant challenges, ASOCT is an essential modality for guiding surgical decision-making regarding AGV explantation versus retention and subsequent DSAEK.

## Introduction

Ahmed glaucoma valve is a pressure-sensitive, unidirectional valve mechanism crucial for controlling elevated intraocular pressure (IOP) in refractory glaucoma cases where medical management, laser procedures, or conventional filtering surgery has failed or is likely to fail. AGV may also be utilized as a primary surgical intervention in select cases. However, corneal decompensation represents a significant complication associated with these devices, with reported incidence ranging from 3.3% to 27% across various studies [1-3].

Multiple mechanisms have been proposed for AGV-related endothelial damage and subsequent corneal decompensation, including direct or intermittent tube-endothelial contact, chronic IOP elevation, and prolonged exposure to antiglaucoma medications. Previous studies have shown that tubes closer to the cornea are associated with greater endothelial cell loss [4,5].

ASOCT has emerged as a valuable tool for anterior segment evaluation. In cases of AGV-related corneal decompensation, it enables precise measurement of critical parameters, including the tube-endothelial distance, the tube-iris distance, corneal thickness, and stromal scarring. These measurements provide crucial information for surgical planning, particularly when considering DSAEK. We present our experience with ASOCT-guided management of 11 eyes with AGV-related corneal decompensation and propose a systematic decision-making protocol for AGV retention versus explantation.

## Materials and methods

This study adheres to the tenets of the Declaration of Helsinki, and approval of the Institutional Ethics Committee was obtained. Written informed consent was obtained from all patients. This prospective interventional study included patients who developed corneal decompensation following primary AGV implantation done at least 6 months or earlier for primary or secondary glaucoma. Patients who underwent AGV implantation within the last 6 months of presentation with corneal decompensation were excluded from the study. Patients for whom ASOCT imaging was not possible for any reason were also excluded from the study.

**Fig. 1:**
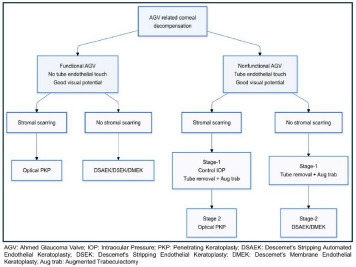
Protocol for management of AGV-related corneal decompensation

All patients were evaluated and managed according to our suggested protocol for AGV-related corneal decompensation (Fig. 1). The comprehensive evaluation included uncorrected visual acuity (UCVA) and best-corrected visual acuity (BCVA), IOP measurement without medications, fundus evaluation to document glaucomatous optic neuropathy, and specular microscopy. Anterior segment evaluation was performed using 5-line raster mode of Cirrus HD-OCT (Carl Zeiss Meditec AG, Jena, Germany).

ASOCT measurements included corneal thickness, assessment of stromal edema or scarring, and tube tip to endothelial (TTE) distance. For TTE measurement, the patient looked at the fixation light while the upper eyelid was gently retracted without globe pressure. The anterior segment scan protocol was used after aligning the scan line perpendicular to the AGV tube axis at its tip, and measurements were taken with calipers. A single examiner recorded all measurements on ASOCT to ensure consistency.

Based on our management protocol (Fig. 1), patients with no stromal scarring, no tube endothelial touch, TTE more than 150 μm, corneal thickness less than 900 μm, functional AGV with good IOP control, and good visual potential underwent DSAEK without AGV modification. For patients with tube-endothelium touch, TTE distance less than 150 μm, or non-functional AGV, a two-stage procedure was performed. The first stage involved AGV tube removal and augmented trabeculectomy. When appropriate IOP control was achieved, DSAEK was performed as the second stage after a minimum period of two months.

Postoperative follow-up included assessment of BCVA, slit-lamp biomicroscopy, ASOCT imaging, and specular microscopy.

## Results

The study cohort comprised 11 eyes from 10 patients (6 males and 5 females), including 1 female patient with bilateral involvement. According to the records, all patients had undergone primary AGV implantation between 2007 and 2013. The underlying diagnosis was primary open-angle glaucoma in nine patients and chronic angle-closure glaucoma in two patients. None of these patients had neovascular or uveitic glaucoma (Table 1-3).

**Table 1:** Preoperative and postoperative data for eleven patients with AGV-related corneal decompensation who underwent DSAEK - preoperative clinical parameters

SER	Age (years)	Gender	Preop-BCVA	Preop IOP (mmHg)	CCT (μm)
1	59	F	CFCF	15	660
2	53	M	HMCF	16	712
3	62	F	CFCF	14	764
4	52	M	HMCF	16	822
5	69	M	CFCF	14	689
6	81	M	CFCF	13	771
7	65	M	1/60	22	788
8	59	F	CFCF	36	741
9	72	M	HMCF	32	768
10	54	F	HMCF	18	837
11	57	F	CFCF	37	896

BCVA = Best Corrected Visual Acuity; IOP = Intraocular Pressure; CCT = Central Corneal Thickness

**Table 2:** Preoperative and postoperative data for eleven patients with AGV-related corneal decompensation who underwent DSAEK - ASOCT parameters, AGV status, and surgical approach

SER	TTE (μm)	Stromal Scarring*	Year of AGV Implantation	AGV Functional Status	Surgical Approach (One or Two Stage)
1	172	No	2013	Yes	One
2	182	No	2012	Yes	One
3	202	No	2013	Yes	One
4	198	Minimal	2011	Yes	One
5	230	No	2012	Yes	One
6	190	No	2013	Yes	One
7	76	No	2007	Yes	Two
8	156	No	2013	No	Two
9	0	No	2008	No	Two
10	92	No	2010	No	Two
11	42	No	2012	No	Two

TTE = Tube Tip to Endothelium distance; ASOCT = Anterior Segment Optical Coherence Tomography; AGV = Ahmed Glaucoma Valve

**Table 3:** Preoperative and postoperative data for eleven patients with AGV-related corneal decompensation who underwent DSAEK - surgical details and postoperative outcomes

SER	AGV Explant	Aug Trab	DSAEK Done	DSAEK Lenticule Thickness	Postop IOP (mmHg)	Postop BCVA
1	No	No	Yes	112	17	20/80
2	No	No	Yes	122	16	20/120
3	No	No	Yes	98	16	20/60
4	No	No	Yes	132	18	20/120
5	No	No	Yes	102	16	20/80
6	No	No	Yes	98	16	20/40
7	Yes	Yes	Yes	140	20	20/30
8	Yes	Yes	Yes	68	14	20/60
9	Yes	Yes	Yes	142	14	20/120
10	Yes	Yes	Yes	148	22	20/200
11	Yes	Yes	Yes	204	12	CFCF

BCVA = Best Corrected Visual Acuity; IOP = Intraocular Pressure; TTE = Tube Tip to Endothelium distance; AGV = Ahmed Glaucoma Valve; Aug Trab = Augmented Trabeculectomy; DSAEK = Descemet Stripping Automated Endothelial Keratoplasty

**Fig. 2:**
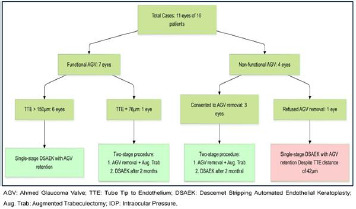
Patient Flow Diagram

Among the eleven eyes studied, seven had functional AGV with well-controlled IOP, while four had non-functional AGV with elevated IOP. Of the seven eyes with functional AGV, six demonstrated TTE distances greater than 150 μm and underwent DSAEK in a single stage, with AGV retained in situ. One patient with functional AGV but a TTE distance of 76 μm underwent a two-stage procedure: AGV explantation with augmented trabeculectomy in stage 1, followed by DSAEK 2 months later in stage 2 (Fig. 2).

**Fig. 3:**
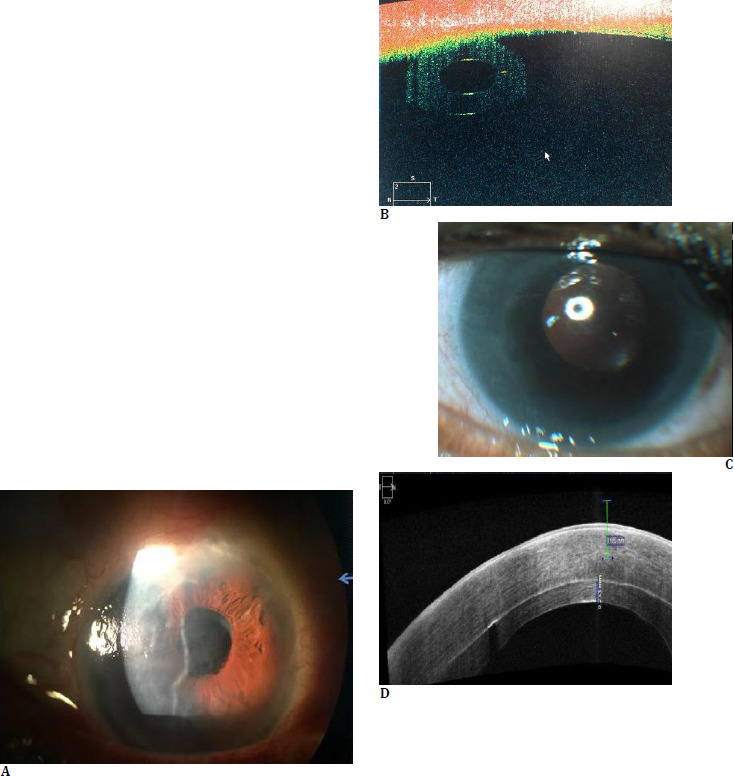
Pre- and post-operative photographs of a patient with tube-endothelial touch (TTE=0). A. Preoperative slit-lamp photograph showing corneal decompensation with a distinct demarcation line (black arrowhead) extending from the region of tube touch; B. Preoperative ASOCT demonstrating tube-endothelial touch with TTE=0; C. Post-operative slit-lamp photograph following AGV explantation and DSAEK showing clear cornea with well-attached graft; D. Post-operative ASOCT confirming optimal graft attachment

Of the four patients with non-functional AGV, three consented to AGV explantation. One of these three patients had documented tube-endothelial touch with TTE=0 (Fig. 3A-D). These patients underwent augmented trabeculectomy following AGV removal, with subsequent DSAEK performed after achieving IOP control. The fourth patient refused explantation of AGV despite a non-functional status, but consented to DSAEK.

**Fig. 4:**
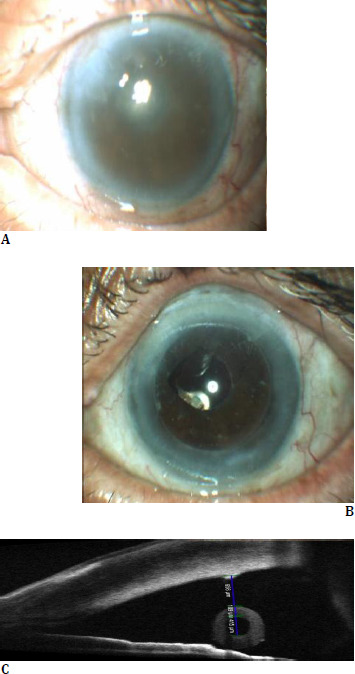
Pre- and post-operative photographs of a patient with adequate TTE distance managed with single-stage DSAEK. A. Preoperative slit-lamp photograph showing diffuse corneal edema and decompensation; B. Post-operative slit-lamp photograph demonstrating clear cornea with well-attached DSAEK graft (black arrowhead indicating tube position); C. Preoperative ASOCT showing safe TTE distance (>150μm)

None of the patients had a very long AGV tube, which would have required trimming. Preoperative corneal thickness measurements in all patients ranged from 689 to 896 μm. No significant stromal scarring was observed in the visual axis on preoperative ASOCT imaging in any patient. The DSAEK lenticules were prepared using a microkeratome and an artificial anterior chamber (SLc, Gebauer, Neuhausen, Germany), with thicknesses ranging from 68 μm to 204 μm (Fig. 4A-C).

**Fig. 5:**
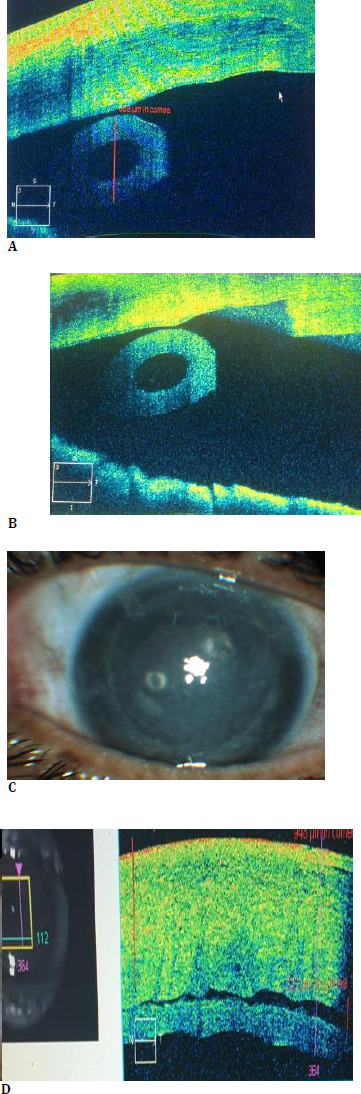
Pre- and post-operative photographs of a patient with inadequate TTE distance (42 μm) who developed complications. A, B. Preoperative ASOCT scans demonstrating critically low TTE distance of 42 μm; C. Post-DSAEK slit-lamp photograph showing persistent corneal edema with visible tube-graft touch (black arrowhead); D. Four weeks post-operative ASOCT showing interface fluid collection and graft detachment

The postoperative course was uneventful in all but one case. The patient, who declined AGV removal despite non-functional status and a TTE distance of 42 μm, developed an interface fluid collection approximately 1 month after DSAEK. This complication, attributed to the tube tip contacting the graft edge, did not resolve with conservative management and necessitated repeat DSAEK (Fig. 5A-D). In all other patients, BCVA improved significantly, and IOP remained well controlled throughout the follow-up period.

## Discussion

Tube shunt implantation effectively reduces IOP in eyes with uncontrolled glaucoma, but it can lead to significant endothelial cell loss and corneal decompensation in a considerable number of patients [6,7]. In our case series, two major challenges were anticipated. Firstly, the decision regarding tube removal was complex, as closure of the port of entry into the anterior chamber is difficult and potentially leaky, raising concerns about adequate IOP control with augmented trabeculectomy after AGV explantation. We also faced the dilemma of whether complete plate removal was necessary or whether tube removal alone would suffice, given that plate removal requires extensive dissection and carries a risk of tissue damage due to significant fibrosis around the plate. Since retaining a non-functional AGV tube in the anterior chamber serves no purpose in patients with established corneal decompensation, we removed the tube in all non-functional AGV cases. We performed augmented trabeculectomy as the first stage for IOP control. DSAEK was subsequently performed after a minimum period of two months as a second-stage procedure.

Our second major challenge was determining a safe TTE distance to prevent graft damage from the tube. With no established guidelines in the literature, we considered a preoperative TTE greater than 150 μm safe, accounting for the typical DSAEK lenticule thickness and the anticipated reduction in corneal edema following successful surgery.

We calculated TTE distance instead of the tube corneal angle (T-C angle), as was done in some earlier published studies, because we believe it is a direct measure of the proximity of the AGV to the endothelium nearer the visual axis and can be important in post-DSAEK patients for assessing the proximity of the DSAEK lenticule to the AGV tube [8]. This measurement proved particularly valuable in predicting potential complications and guiding surgical decision-making.

In our series of 11 cases, one patient had a tube that touched the endothelium, necessitating explantation. In two other patients, TTE distances were critically low (42 and 76 μm), and both were advised of AGV removal. However, one patient declined removal and subsequently developed graft detachment at 1 month due to tube-graft edge contact (Fig. 5 A-D). Two other patients with non-functional AGV underwent explantation despite TTE distances exceeding 150 μm, followed by augmented trabeculectomy and successful DSAEK. The three patients with functional AGV and adequate TTE distances who underwent DSAEK alone showed uneventful postoperative courses.

While DSAEK in eyes with AGV presents significant challenges, success rates are comparable to those of penetrating keratoplasty in previous studies [9]. Our experience suggests that these outcomes can be further improved through appropriate patient selection and accurate decision-making regarding AGV retention or explantation, in accordance with our protocol (Fig. 1). ASOCT parameters proved invaluable in guiding our surgical approach, particularly in determining the necessity of tube removal and predicting potential complications.

The management of the patient who declined AGV removal despite a non-functional tube and critically low TTE distance provided valuable insights. The subsequent graft detachment in this case reinforced the importance of following our protocol’s recommendations regarding tube removal when indicated. Regular ASOCT evaluation of AGV patients could potentially help identify tube-endothelial proximity issues early, allowing intervention before corneal decompensation develops.

## Conclusion

Performing DSAEK in patients with AGV-related corneal decompensation presents significant surgical challenges, but careful preoperative planning guided by ASOCT can lead to successful outcomes. ASOCT serves as an essential tool in guiding decisions about AGV explantation versus retention and subsequent DSAEK planning. To the best of our knowledge, this systematic approach has not been previously published in the literature. It may have broader implications for performing lamellar corneal surgery in patients with prior AGV implantation. While our sample size is relatively small, more studies with larger cohorts are needed to further refine our suggested protocol for managing patients with AGV-related corneal decompensation and to determine a safe TTE distance.

## Data Availability

All relevant data are included within the manuscript and its supporting information files.

## Presentation at a meeting

Nil.
